# SHIP prevents metastasis

**DOI:** 10.18632/aging.100964

**Published:** 2016-05-16

**Authors:** Gerald Krystal, Melisa J Hamilton, Kevin L Bennewith

**Affiliations:** Department of Integrative Oncology, British Columbia Cancer Agency Research Centre, Vancouver, BC, Canada

**Keywords:** SHIP, PI3K, metastasis

The Src-homology-2-containing inositol-5′-phosphatase, SHIP (also known as SHIP1), is a tumor suppressor that negatively regulates the phosphatidylinositol 3-kinase (PI3K) pathway by hydrolyzing the PI3K-generated second messenger phosphatidylinositol-3,4,5-triphosphate (PIP_3_) to PI-3,4-P_2_. SHIP expression is restricted primarily to hematopoietic cells, but has also been reported in mesenchymal stem cells (MSCs) and osteoblasts [1]. SHIP levels can be reduced by inactivating mutations, single nucleotide polymorphisms, or miR-155 activity [2], and SHIP expression decreases in the aging MSC compartment of murine bone marrow, skewing hematopoiesis toward production of myeloid cells [1] and potentially leading to the development of myeloproliferative syndromes or myeloid neoplasms with age. It is unknown whether SHIP levels change during the aging process in hematopoietic cells, although aberrant PI3K activity in human neutrophils increases with age, potentially via reduction in SHIP, and negatively impacts neutrophil migration and function [3].

While reduction of SHIP has been associated with the development of leukemias and lymphomas[4], the potential role of SHIP in solid tumor growth has received very little attention to date, likely because most researchers believe neoplasms derived from non-hematopoietic tissues should not be affected by SHIP status. However, there is a great deal of evidence that normal cells in the stroma (e.g., fibroblasts, endothelial cells, inflammatory cells) have a profound influence on the development and growth of solid primary tumors and tumor metastases. Stephen Paget's seed-and-soil hypothesis identified the soil in distant tissues as a key limitation of metastatic tumor growth, and recent evidence suggests that inflammatory cells may act as fertilizer for the metastatic soil, with aggregates of normal cells (often derived from the bone marrow) creating permissive niches that support future metastatic tumor growth in tissues [5]. Cells that suppress the immune response against tumor cells may be particularly important, with myeloid-derived suppressor cells (MDSCs), alternatively-activated M2 macrophages (Mϕs), and regulatory T cells (Tregs) often being elevated in tissues that support metastatic tumor growth. Expansion and activity of each of these immune suppressive cell types are known to be restricted by SHIP [4] and it stands to reason that loss of SHIP may lead to increased immune suppressive cell accumulation and activity in tissues, suppressed T-cell mediated anti-tumor immunity, and therefore increased metastatic tumor growth. Support for this concept comes from studies in which SHIP has been deleted in various mouse strains.

Specifically, deleting SHIP from C57BL/6 mice leads to runted mice that overproduce myeloid cells and die at 12-16 weeks of age from massive myeloid cell infiltration of the lungs [4]. This myeloid infiltrate includes elevated numbers of hyper-responsive mast cells (leading to increased histamine and T_H_2 cytokines/chemokines in the lungs). SHIP−/− C57BL/6 mice are both M2 and T_H_2 skewed, in part due to hyperactive basophils that constitutively produce IL-4. Loss of SHIP dramatically increases myelopoiesis, which forces erythropoiesis out of the bone marrow and into the spleen, causing splenomegaly. In addition, SHIP−/− C57BL/6 mice possess an increased number of hyper-resorptive osteoclasts and reduced numbers of osteoblasts [1], and consequently suffer from severe osteoporosis. A critical feature of SHIP−/− C57BL/6 mice is that their myeloid progenitors are significantly more responsive to low levels of cytokines, growth factors, and chemokines than their wild-type counterparts, which explains why SHIP−/− C57BL/6 mice display enhanced myelopoiesis, with overproduction of granulocytes, mast cells, and M2-skewed Mϕs. In keeping with the presence of M2-skewed Mϕs, we observed enhanced growth of Lewis lung carcinoma primary tumours in SHIP−/− C57BL/6 mice compared to wild-type mice [4].

We recently reported that deleting SHIP in BALB/c mice results in a much milder pathology than deleting SHIP in C57BL/6 mice [6]; this was surprising since BALB/c mice are intrinsically more M2/T_H_2 skewed. Because of the milder pathology, SHIP−/− BALB/c mice live a normal lifespan, without evidence of overt osteoporosis or wasting, and display only mild pulmonary inflammation, alveolar wall thickening and myeloid hyperplasia. However, these mice do possess M2-skewed Mϕs that are significantly more immunosuppressive than Mϕs from wild-type mice, and we have shown that highly suppressive M2 Mϕs within the lungs promote metastatic tumour growth [7]. In keeping with these data, challenging SHIP−/− BALB/c mice with metastatic 4T1 mammary carcinoma cells causes the mice to develop red and inflamed ears, paws and tails, dramatically lose weight, and die from necrohemorrhagic inflammatory pulmonary disease within 14 days of tumour implantation. Moreover, unlike tumor-free SHIP−/− BALB/c mice, 4T1 tumor-bearing SHIP−/− BALB/c mice possess MDSCs that express arginase I and are far more immuno-suppressive than their wild-type counterparts. Importantly, while primary 4T1 tumor growth is not significantly different in wild-type and SHIP−/− BALB/c mice, there are 7.5-fold more metastatic tumor cells in the lungs of mice lacking SHIP. Our work demonstrates that SHIP represses the metastasis of mammary tumors in BALB/c mice by restricting the development and immunosuppressive properties of myeloid cells [6].

If SHIP expression and activity are indeed reduced during the aging process, the concomitant elevation in PI3K signaling in hematopoietic cells may predispose people to reduced neutrophil function, myelo-proliferative disorders, leukemias, and lymphomas. Our data suggest that reduction of SHIP also increases the risk of developing solid tumors and predisposes cancer patients to metastatic disease. We have identified SHIP as a new player in solid tumor metastasis, and support future work to test activators of SHIP for the management of metastatic cancer.
